# Mallard hindlimbs locomotion system respond to changes in sandy ground hardness and slope

**DOI:** 10.1038/s41598-024-66181-z

**Published:** 2024-07-05

**Authors:** Dianlei Han, Lizhi Ren, Hairui Liu, Jinrui Hu, Guoyu Li

**Affiliations:** 1 School of Agricultural Engineering, Jiangsu University, 301 Xuefu Road, Zhenjiang, 212013 Jiangsu China; 2 Key Laboratory of Modern Agricultural Equipment and Technology (Jiangsu University), Ministry of Education, Zhenjiang, 212013 China; 3 School of Mechanical Engineering, Shanghai Dianji University, Shanghai, 201306 China

**Keywords:** Mudflat ground, Mallard, Hindlimb locomotion, Hardness, Slope, Leg-foot coordination, Animal behaviour, Biomechanics

## Abstract

Mallards inhabit soft grounds such as mudflats, marshes, and beaches, demonstrating remarkable proficiency in traversing these grounds. This adeptness is closely linked to the adjustments in the operation of their hindlimbs. This study employs high-speed videography to observe postural adjustments during locomotion across mudflats. Analysis of spatiotemporal parameters of the hindlimbs reveals transient and continuous changes in joints (tarsometatarso-phalangeal joint (TMTPJ), intertarsal joint (ITJ), knee, and hip) during movement on different ground hardness and slope (horizontal and uphill). The results indicate that as the stride length of the mallard increases, its speed also increases. Additionally, the stance phase duration decreases, leading to a decrease in the duty factor. Reduced ground hardness and increased slope lead to delayed adjustment of the TMTPJ, ITJ, and knee. Mallards adjust their stride length by augmenting ITJ flexion on steeper slopes, while reduced hardness prompts a decrease in TMTPJ flexion at touch-down. Additionally, the hip undergoes two brief extensions during the stance phase, indicating its crucial role in posture adjustment and propulsion on uphill grounds. Overall, the hindlimb joints of the mallard function as a whole musculoskeletal system, with each joint employing a distinct strategy for adjusting to adapt to various ground conditions.

## Introduction

Mudflats are complex sand grounds frequently inundated by tides, consisting of various types of grounds including naturally soft sand ground, uphill sand ground, and hard sand ground continuously eroded by river. Animals moving on these grounds frequently encounter challenges such as sinking, slipping, and poor traversal capability. Mallards, as amphibious creatures, predominantly inhabit softer grounds like mudflats and beaches, exhibiting exceptional maneuverability on these grounds. The adaptive modifications in the webbed feet and hindlimb postures of mallards are pivotal for navigating various sand grounds.

Scholars have conducted extensive research on the spatiotemporal parameters of animal locomotion and the effect of diverse factors on these locomotion. The analysis of animal hindlimbs frequently includes the examination of spatiotemporal parameters during locomotion, with locomotion speed often used as a standard metric for assessing locomotive efficiency. Commonly, birds adjust their speed by altering either stride frequency or stride length. Studies have shown that smaller birds, such as quails, guinea fowls, mallards, and barnacle goose (*Brante leucopsis*), predominantly modify their stride length, while larger birds such as ostriches and other smaller birds such as kiwis both primarily increase their stride frequency^1–4^. In parallel, the adult salamander (*Dicampodon tenebrosus*) adjusts its speed by varying stride length^5^, and the American alligator (*Alligator mississippiensis*) alters its speed through changes in hindlimb posture^6^. Black-billed magpies display distinct walking, running, and jumping patterns through differential adjustments in hindlimb joint angles^7,8^.

Quantitative studies have also been conducted on the hindlimb kinematics of bipedal birds such as quails (*Coturnix japonica*), guinea fowls, northern lapwings, and white storks (*Ciconia ciconia*), as well as lizards (*Dipsosaurus dorsalis*)^9–14^. Different lizard species exhibit varied joint angles when sprinting at maximum speeds^15^. Some animals, such as ostriches, not only exhibit hindlimb locomotion in the sagittal plane but also perform adduction and abduction in a three-dimensional space^16^. The aforementioned scholars provide valuable methodologies and perspectives for studying mallard hindlimb locomotion, noting kinematic differences among various species. Although researchers have explored the spatiotemporal parameters of indoor mallard locomotion^17^, limited research has been conducted on outdoor locomotion in natural land environments. The typical waddling of a duck requires non-sagittal movements. However, there has been no quantification of non-sagittal locomotion in the hindlimbs of webbed-foot birds like the mallard.

Substrates significantly effect animal locomotion, with diverse coping strategies emerging in response to different ground conditions. Variation in the hindlimb postures of animals enables them to leverage their innate advantages when navigating through different environments. In the natural environment, animals face numerous ground challenges, such as differences in softness, slope, particle size, and humidity. Lizards, for instance, adjust stride length and stride frequency in response to changes in medium particle size^18^. Zebra-tailed lizards (*Callisaurus draconoides*) increase their stride frequency on fluidized particulate ground as opposed to solid ground^19^. Sea turtles (*Caretta caretta*) navigate sand grounds by solidifying particles beneath their flippers, reducing slippage^20^. Humans, when moving on soft sand ground, adapt by increasing hip and knee range of motion to counter challenges such as sand subsidence^21^. Substrate humidity also plays a role in animal locomotion performance^22^. Certain animals, such as mallards and American eels (*Anguilla rostrata*), exhibit remarkable adaptability in various environments. These aquatic animals adjust their locomotion postures in accordance with their habitats' physical characteristics^23,24^. The effect of slope on hindlimb motion has been explored in both *Dipsosaurus dorsalis* and German Shepherd Dogs^25,26^. Previous studies have explored animal hindlimb postures and the adaptive adjustments they make under different conditions, showcasing their unique locomotion advantages. However, conducting most experiments indoors may potentially limit animals' expression of natural locomotion behaviors.

Recent studies have focused on mallards' webbed foot posture changes on various sand grounds^17,27^, yet detailed research into webbed-foot birds hindlimb postural adaptations in natural mudflat environments remains unexplored. Consequently, this research examines three types of natural flat sand ground, natural uphill sand ground, and hard uphill sand ground. These mudflat conditions offer excellent natural grounds for researching animal hindlimb kinematics and response strategies. This investigation provides a novel perspective on animal hindlimb kinematics, particularly in understanding how hindlimb locomotion respond to variations in ground hardness and slope. It is crucial to understand the effect of mudflat ground's hardness and slope on the hindlimb posture of mallards. Analyzing the active adaptation strategies and functionalities of mallard hindlimbs can provide theoretical insights for designing locomotion mechanisms on mudflats.

## Materials and methods

### Animal ethic statement

The Animal Protection and Use Committee of Jilin University, China, approved the living and experimental conditions of the samples (reference No. SY202206100). The experiments in this study comply with the current laws of China. This study did not involve any animal capture or sampling of blood or tissue. During the experiment, mallards actively participated in the walking process and if any mallard exhibited abnormal locomotion, the experiment was immediately halted. The mallards were housed in custom-designed duck cages, ensuring their natural and healthy living conditions by providing adequate water and nutrition. After the experiment was completed, mallards were returned to the farm healthy and safe. It has been confirmed that all experiments were performed in accordance with relevant guidelines and regulations.

### Animals

**Figure 1:**
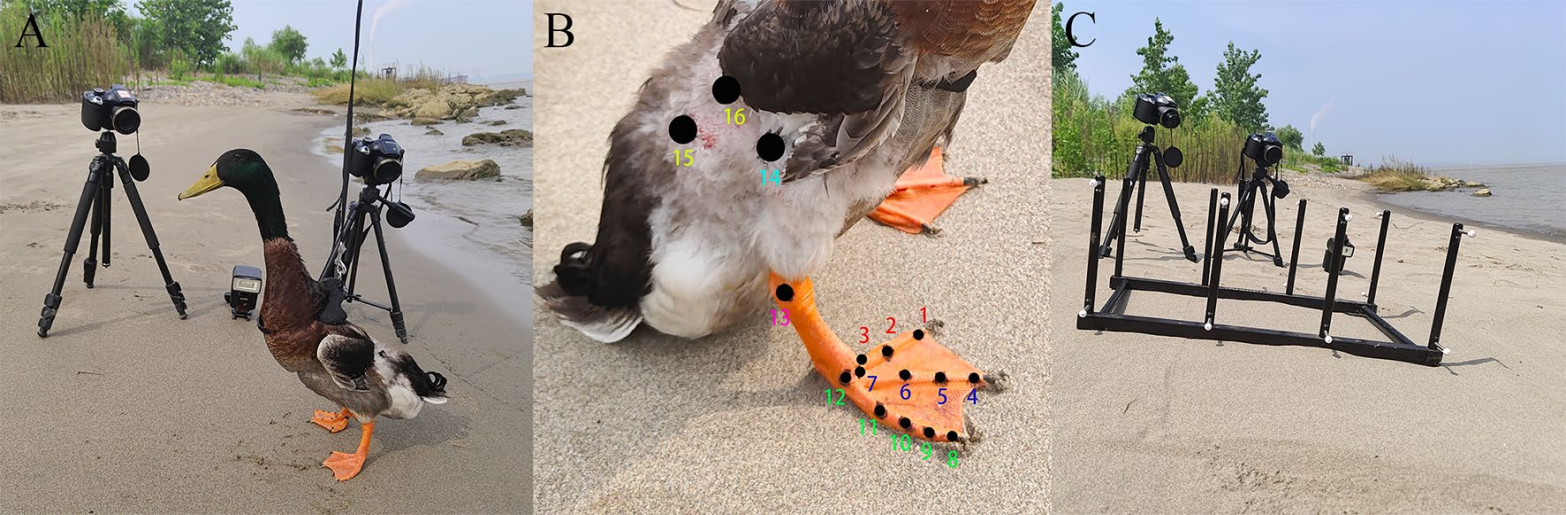
Experimental environment and specific location of marking points. (**A**) Diagram of the experimental process; (**B**) schematic of the specific marking point locations; (**C**) equipment layout (illustrating the natural uphill sand ground with a calibration rack).

Four male mallards, aged 24 months, were selected from a specialized breeding farm in Zhejiang Province, China, for this study, as illustrated in Fig. 1A. Their average weight was 1453.75 ± 59.94 g (expressed as mean ± standard deviation of body weight). In order to enhance their adaptation for the experiment, the mallards underwent a 2-week training regimen on sand ground, with sessions four times a week, each lasting approximately 30 min. To prevent escape during testing and to facilitate clear recording by high-speed cameras, selective trimming of wing feather tips was carried out, ensuring no hindrance to the right hindlimb locomotion of the mallards. Traditional adhesive markers, deemed too cumbersome and likely to detach from the mallard's toe joints, were replaced with a black marker pen. This alteration has facilitated natural movements of the mallard hindlimb, while also allowing for the capture of clear and precise recordings of joint points.

As shown in Fig. 1B, a total of 16 points were marked on the right hindlimb of each mallard for accurate tracking. The second toe of the mallard had three markers in total: the dorsal ridge of the toenail (marker 1), interphalangeal joints of the first and second phalanx (marker 2), and the second tarsometatarso-phalangeal joint (TMTPJ) (marker 3). The third toe had four markers, including the dorsal ridge of the toenail (marker 4), interphalangeal joints of the second and third phalanx (marker 5), interphalangeal joints of the first and second phalanx (marker 6), as well as the third TMTPJ (marker 7). The fourth toe had five markers: the dorsal ridge of the toenail (marker 8), interphalangeal joints of the third and fourth phalanx (marker 9), interphalangeal joints of the second and third phalanx (marker 10), interphalangeal joints of the first and second phalanx (marker 11), as well as the fourth TMTPJ (marker 12). Additionally, markers were placed at the intertarsal joint (ITJ) (marker 13), knee (marker 14), hip (marker 15) and craniolateral preacetabular part of the ilium (marker 16).

### Experimental design

The experimental location was set on a riverbank in Shiye Town, Zhenjiang, China, which belongs to a completely natural environment. The habitats along the riverbank mainly consist of natural flat sand ground, natural uphill sand ground, and hard uphill sand ground. Therefore, we chose to conduct experiments under these three different conditions. The natural flat sand ground was represented by a natural loose horizontal mudflats, whereas the natural and hard uphill sand grounds were selected from natural loose and hard uphill mudflats, respectively, each with a gradient of 8°–10°. All surfaces were leveled with brushes to ensure the smoothness of the sand met the experimental requirements. Table 1 displays the particle size distribution for both the natural and hard sand grounds. Each mallard was guided to move bipedally in the different ground environments using a duck leash. In each environment, each mallard was required to effectively repeat the experiment at least ten times. As shown in Fig. 1C, kinematic analysis necessitated the use of at least two high-speed cameras (Casio Exilim EX-FH25, Tokyo, Japan), recording at 120 Hz/s and positioned at angles greater than 60° to each other for accurate three-dimensional (3D) space restoration^28,29^. The cameras were placed on the right front and right sides of the experimental field to ensure optimal coverage of the mallards during locomotion. Each camera had a pixel size of 640 × 480. A 16-point calibration frame was used for 3D coordinate calibration, requiring the calibration angle to match the motion video angle. Therefore, the camera positions were kept fixed until the motion video recording was completed before concluding the experiment.

**Table 1:** Particle size distribution proportion of natural sand and hard sand.

Particle size (mm)	Hard sand		Natural sand	
	Weight (g)	Proportion (%)	Weight (g)	Proportion (%)
> 0.355	0.22	0.02%	0.21	0.05%
0.250–0.355	0.92	0.09%	0.84	0.19%
0.180–0.250	40.00	4.05%	37.69	8.63%
0.150–0.180	534.00	54.11%	302.00	69.13%
0.125–0.150	200.00	20.27%	70.00	16.02%
0.090–0.125	185.50	18.80%	23.00	5.27%
0.075–0.090	0.62	0.06%	0.10	0.02%
< 0.075	25.69	2.60%	3.00	0.69%
Total	986.95	100.0%	436.84	100.0%

### Data processing

High-speed cameras captured videos that were filtered to select four mallards exhibiting uniform motion speeds and optimal conditions. These videos were then imported into kinematic software (Simi Reality Motion Systems GmbH, Unterschleissheim, Germany) for data processing. The software tracks marker points on the right hindlimb of each mallard, and joint angles intended to represent non-sagittal flexion or extension of the mallard's motion are obtained through 3D space recovery and calculation using Simi-Motion software. At least six complete stride cycles were analyzed for each mallard across different sand ground. This process facilitated the acquisition of both instantaneous and continuous joint angle changes in the TMTPJ, ITJ, knee, and hip of the hindlimb. Subsequently, the processed data was organized using Excel (Microsoft, Redmond, WA, USA). Analyzing instantaneous changes in each joint angle involved selecting moments such as touch-down, mid-stance, lift-off, and mid-swing. The continuous assessment of joint angle involved normalizing the entire stride cycle based on average locomotion time, thus dividing the stride duration into continuous and uniform time intervals. This method yields continuous joint angle variation curves for the mallard, spanning from right foot touch-down (0%) to next touch-down (100%), enabling the analysis of each joint angle's continuous variation pattern. Charts illustrating these findings were generated using Origin Pro 8.5 (OriginLab Corporation, Northampton, MA, USA). A one-way ANOVA was conducted to discern differences across each working condition of the mallard^30,31^. Results were deemed statistically significant at *p* < 0.05.

## Results

### Spatiotemporal parameters and hindlimb locomotion posture of mallards

Table 2 presents the spatiotemporal parameters observed in mallards moving under various conditions. Specifically, stride length, defined as the distance between consecutive touchpoints of the mallard's right hindlimb, is recorded. Observations reveal that on sand grounds with uniform hardness, an increasing slope extends the stance phase duration and increases the duty factor, subsequently leading to reduced speed and shorter stride length. Similarly, on sand ground with a consistent slope, a decrease in ground hardness also prolongs the stance phase and heightens the duty factor, similarly resulting in decreased speed and stride length. Our results indicate that an increase in slope and softer ground lead to a notable decrease in the stride length of mallards. While the swing phase duration remains relatively constant, there is a discernible increase in the stance phase duration across different sand grounds.

**Table 2:** Spatiotemporal parameters of mallards during locomotion on different sand ground.

sand ground	Stance phase Duration (s)	Swing phase Duration (s)	Stride duration (s)	Duty factor	Stride length(m)	Speed (m/s)
Natural flat sand	0.13	0.13	0.26	0.52	0.52	2.09
Natural uphill sand	0.22	0.14	0.36	0.60	0.44	1.32
Hard uphill sand	0.14	0.14	0.28	0.51	0.48	1.86

**Figure 2:**
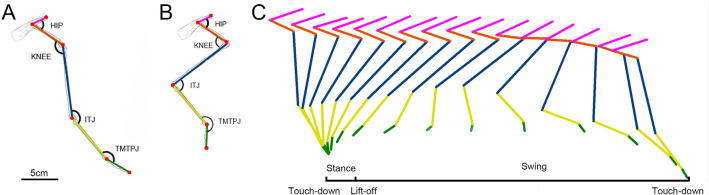
Diagrammatic representation of mallard hindlimb postures during extension and flexion. (**A**) Posture at hindlimb touch-down; (**B**) mid-swing hindlimb posture; (**C**) single-stride cycle hindlimb posture diagram on natural uphill sand ground (with a 1/60 s interval between each line). *TMTPJ* Tarsometatarso-phalangeal Joint, *ITJ* intertarsal joint.

This study focuses on the right hindlimb of mallards, examining postural changes throughout a stride cycle. Similar to other terrestrial birds, mallards adopt a semi-squatting posture. At touch-down, the hindlimb, nearly fully extended, contacts the ground ahead of the body's hip (Fig. 2A). The early stance phase involves hindlimb compression predominantly via the knee and TMTPJ flexion. As stance phase duration progresses, the hindlimbs move backward relative to the body. When the hip advances beyond the toes, the body propels forward; while knee flexion persists in the later stages of the stance phase, hindlimb extension is facilitated through the TMTPJ and ITJ. During the swing phase, knee and ITJ flexion provide ground clearance for the hindlimbs (Fig. 2B). Throughout a stride cycle, the early stance phase sees slight hindlimb compression and increasing toe contact. Later stages of the stance phase involve joint extension, driving the body forward. The early swing phase sees joint flexion lifting the toes off the ground, while later, joints rapidly extend in preparation for the next touch-down (Fig. 2C).

### Instantaneous joint angle

#### TMTPJ

**Figure 3:**
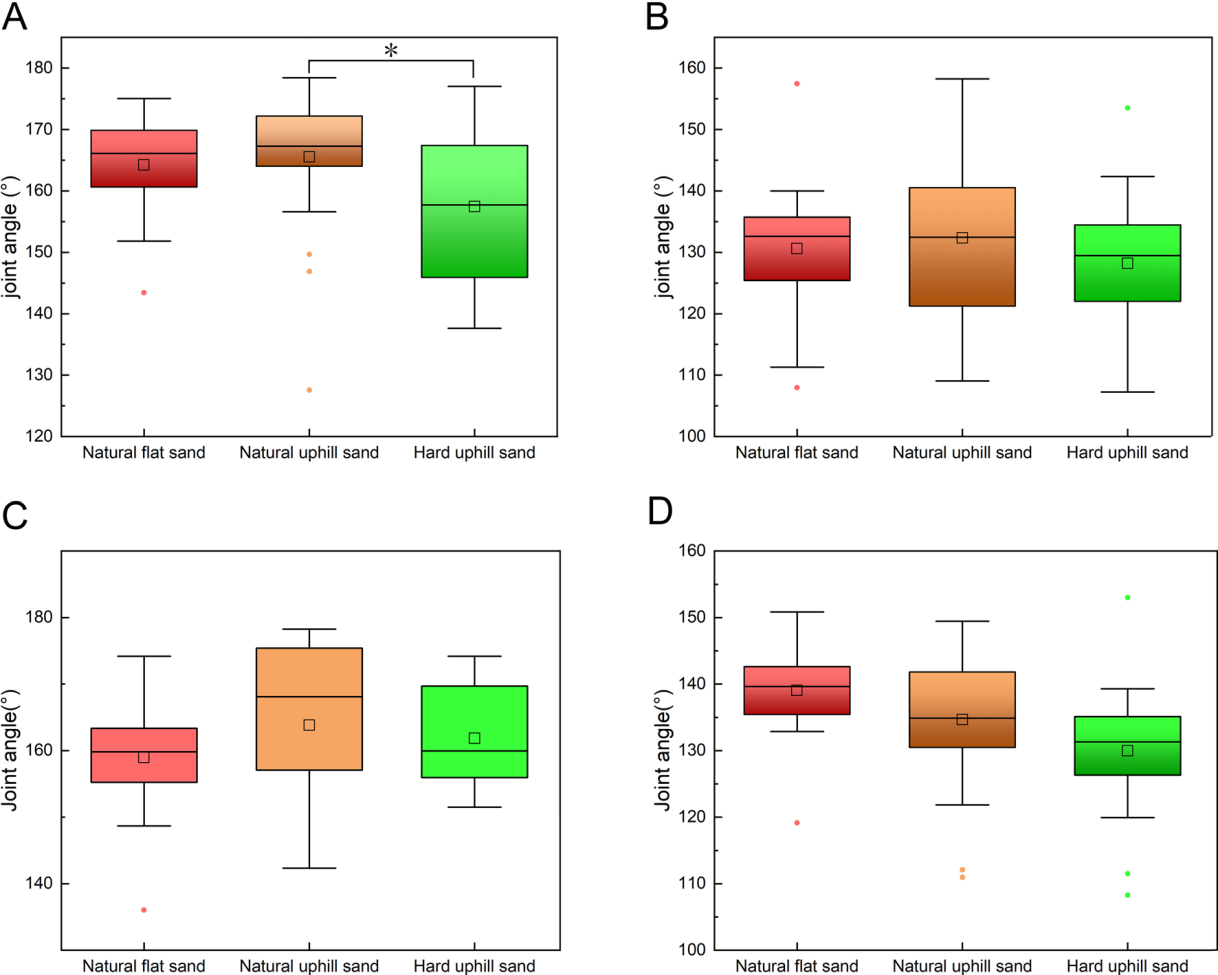
Instantaneous joint angles of the TMTPJ on natural flat sand ground, natural uphill sand ground, and hard uphill sand ground. (**A**) At touch-down; (**B**) at mid-stance; (**C**) at lift-off; (**D**) at mid-swing. Each operating condition involved the analysis of 24 stride cycles. The box plot shows the median, upper and lower quartiles, and highest and lowest values. A hollow rectangle indicates the horizontal mean, and a solid diamond represents outliers. Significant differences identified via Bonferroni's test are indicated with asterisks (p < 0.05).

Figure 3A illustrates a significant difference in the TMTPJ angle at touch-down between natural uphill and hard uphill sand grounds (p = 2.5 × 10^−2^), as determined through one-way analysis of variance. Specifically, the flexion angle of the TMTPJ in mallards is smaller when moving on natural uphill sand ground. Figures 3B–D reveal no significant differences in the TMTPJ angle across natural uphill, natural flat, and hard uphill sand grounds during mid-stance, lift-off, and mid-swing.

#### ITJ

**Figure 4:**
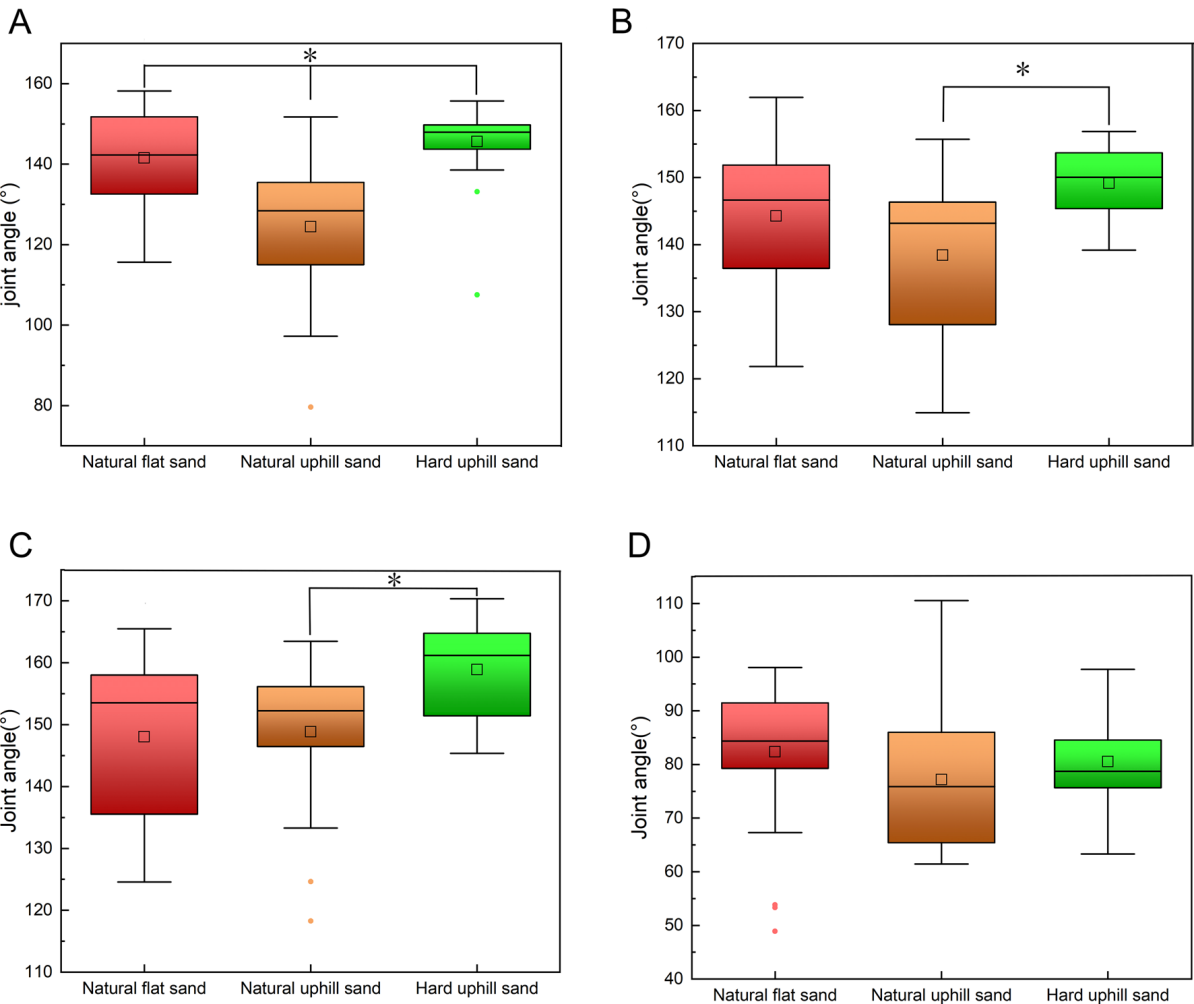
Instantaneous joint angles of the ITJ on natural flat sand ground, natural uphill sand ground, and hard uphill sand ground. (**A**) At touch-down; (**B**) at mid-stance; (**C**) at lift-off; (**D**) at mid-swing. Each operating condition involved the analysis of 24 stride cycles. The box plot shows the median, upper and lower quartiles, and highest and lowest values. A hollow rectangle indicates the horizontal mean, and a solid diamond represents outliers. Significant differences identified via Bonferroni's test are indicated with asterisks (p < 0.05).

Figure 4A reveals, through one-way analysis of variance, that there was a significant difference in the ITJ angle during touch-down between locomotion on natural uphill and flat sand grounds at the touch-down (p = 8.8 × 10^−5^). Additionally, a significant difference exists in the ITJ angle at touch-down between locomotion on natural uphill and hard uphill sand ground (p = 1.6 × 10^−6^). On natural uphill sand grounds, the ITJ exhibits greater flexion during touch-down. As depicted in Fig. 4B, a significant difference is observed in the ITJ angle during mid-stance (p = 2.5 × 10^−2^) between the natural uphill and hard uphill sand grounds, with increased flexion on natural uphill grounds. In Fig. 4C, the difference in ITJ flexion at lift-off between natural uphill and hard uphill sand grounds is significant (p = 6.9 × 10^−3^), with greater flexion observed on natural uphill grounds. However, during mid-swing, there are no significant differences in ITJ angle across natural uphill, natural flat, and hard uphill sand grounds.

#### Knee

**Figure 5:**
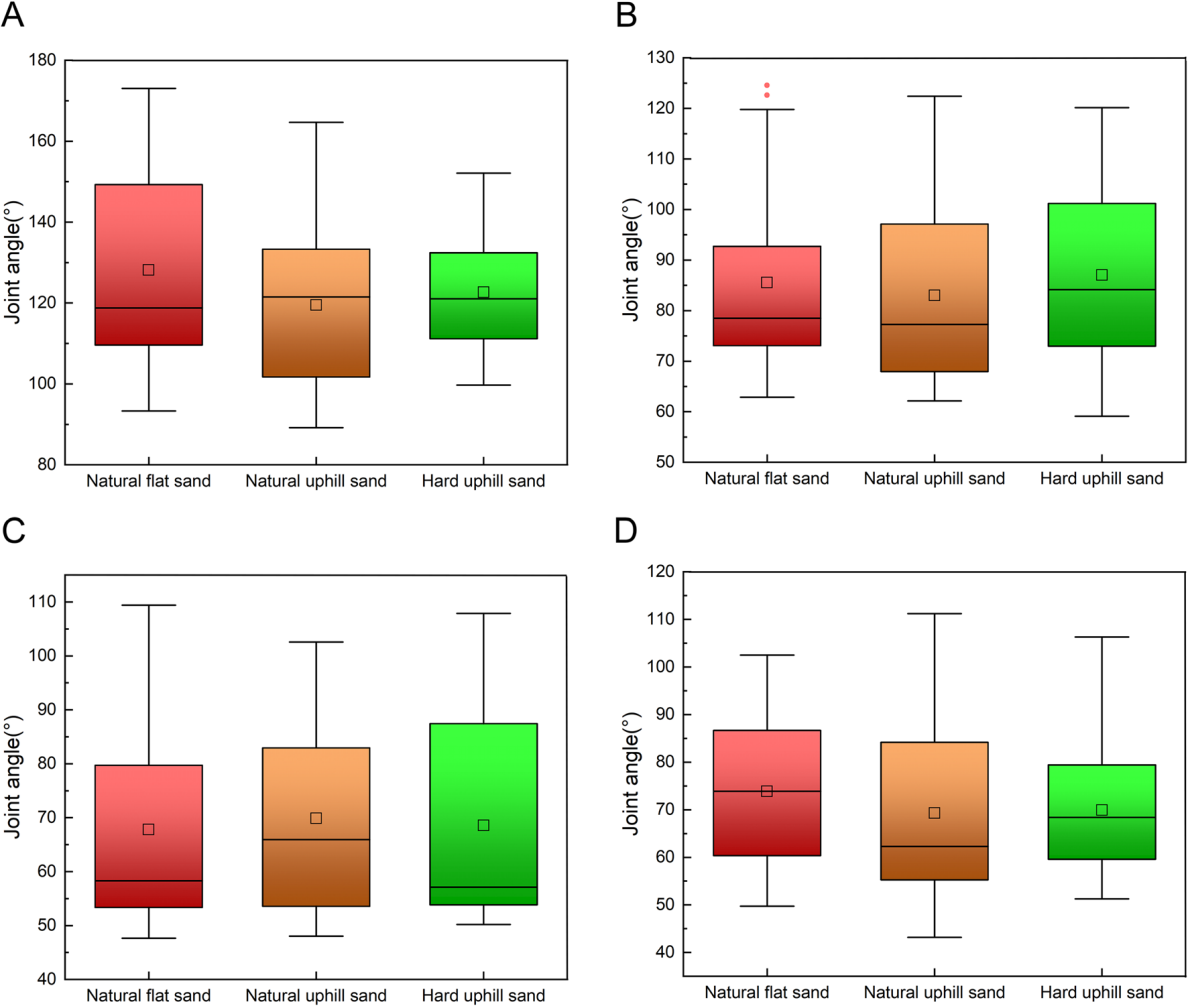
Instantaneous joint angles of the knee on natural flat sand ground, natural uphill sand ground, and hard uphill sand ground. (**A**) At touch-down; (**B**) at mid-stance; (**C**) at lift-off; (**D**) at mid-swing. Each operating condition involved the analysis of 24 stride cycles. The box plot shows the median, upper and lower quartiles, and highest and lowest values. A hollow rectangle indicates the horizontal mean, and a solid diamond represents outliers. Significant differences identified via Bonferroni's test are indicated with asterisks (p < 0.05).

As indicated in Fig. 5A–D, a one-way analysis of variance demonstrates no significant differences in knee angles during touch-down, mid-stance, lift-off, and mid-swing across natural uphill, natural flat, and hard uphill sand grounds.

#### Hip

**Figure 6:**
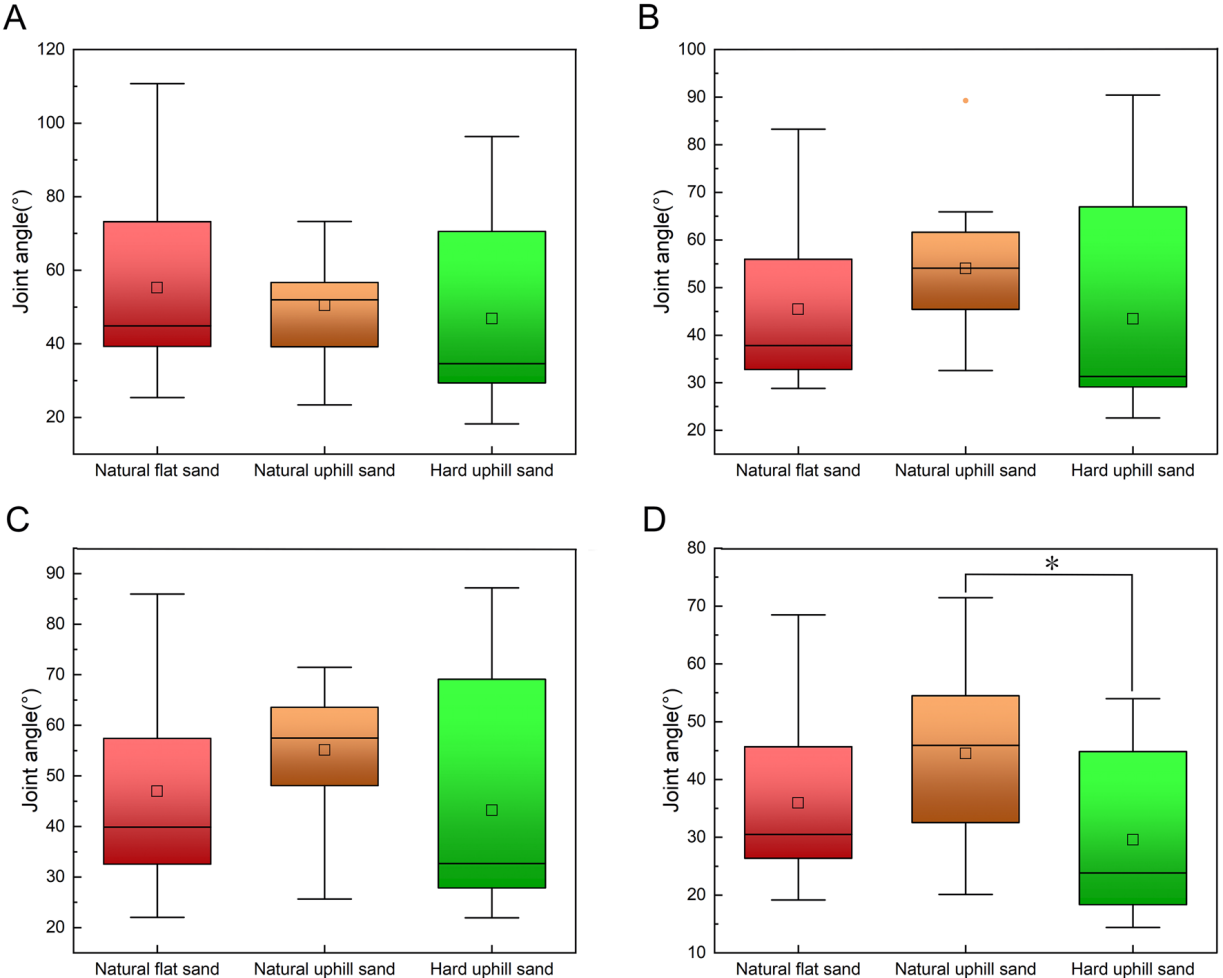
Instantaneous joint angles of the hip on natural flat sand ground, natural uphill sand ground, and hard uphill sand ground. (**A**) At touch-down; (**B**) at mid-stance; (**C**) at lift-off; (**D**) at mid-swing. Each operating condition involved the analysis of 24 stride cycles. The box plot shows the median, upper and lower quartiles, and highest and lowest values. A hollow rectangle indicates the horizontal mean, and a solid diamond represents outliers. Significant differences identified via Bonferroni's test are indicated with asterisks (p < 0.05).

Figure 6A–C illustrates, via a one-way analysis of variance, that there are no significant differences in hip angles during touch-down, mid-stance, and lift-off between locomotion on natural uphill, natural flat, and hard uphill sand grounds. However, Fig. 6D indicates a significant difference in hip angles during mid-swing (p = 1.6 × 10^−6^) between natural uphill and hard uphill sand grounds. On natural uphill sand ground, the hip demonstrates reduced flexion during mid-swing.

In summary, the study's findings reveal that when mallards move on sand grounds with identical slopes but decreasing hardness, the following adaptations occur: there is an increase in the TMTPJ angle at touch-down, a reduction in the ITJ angle at touch-down, mid-stance, and lift-off, and no notable changes in knee angles at any phase. The hip angle exhibits an increase during mid-swing. These results suggest that mallards adjust their TMTPJ and ITJ in response to decreased ground hardness, modify their ITJ during mid-stance and lift-off, and alter their hip joints during mid-swing.

When mallards move on sandy grounds with the same hardness levels, alterations in the ground slope primarily affect the angle of the ITJ. Notably, an elevation in slope leads to a decrease in the ITJ angle at the stance phase. However, this increase in slope does not induce significant changes in the angles of the TMTPJ, knee, and hip throughout any phase of locomotion. This observation suggests that the mallard predominantly adapts its ITJ in response to increased slope conditions, whereas other joints, such as the TMTPJ, knee, and hip, do not exhibit significant differences during the touch-down, mid-stance, lift-off, and mid-swing.

### Continuous joint angles

#### TMTPJ and ITJ

**Figure 7:**
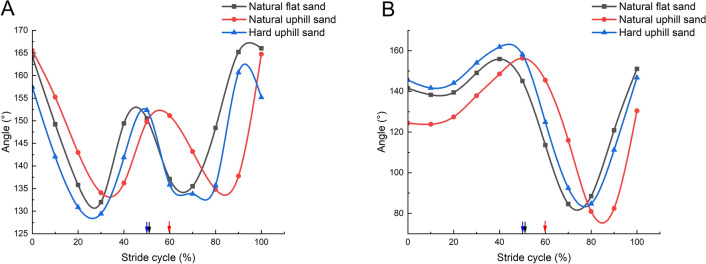
Effect of different sand grounds on continuous joint angles. (**A**) TMTPJ variation pattern effected by locomotion on natural flat sand ground, natural uphill sand ground, and hard uphill sand ground; (**B**) ITJ variation pattern effected by locomotion on natural flat sand ground, natural uphill sand ground, and hard uphill sand ground. (24 stride cycles were analyzed for each condition. Arrows denote the transition from the touch-down to the swing phase.)

Figure 7A illustrates the variation in the TMTPJ angle, which initially decreases and subsequently increases during the stance phase, culminating in a peak resembling the middle of a 'W' shape towards the conclusion of the stance phase. This pattern recurs during the swing phase, with the angle first declining, then rising, before resetting to its initial value at the next touch-down. The continuous variation trend of the TMTPJ angle remains consistent across different grounds. However, a notable rightward shift in this trend is observed when mallards move on natural uphill sand ground. Figure 7B depicts the ITJ angle's dynamics, characterized by an initial decrease followed by an increase during the stance phase. At lift-off, the ITJ angle attains its maximum, then swiftly drops and rises again during the swing phase. The range of ITJ angle variation is less pronounced during the stance phase compared to the swing phase. The continuous variation pattern of the tarsal joint angle exhibits similarity across various grounds, but a distinct rightward shift in this trend is evident when the mallards move on natural uphill sand ground.

#### Knee and hip

**Figure 8:**
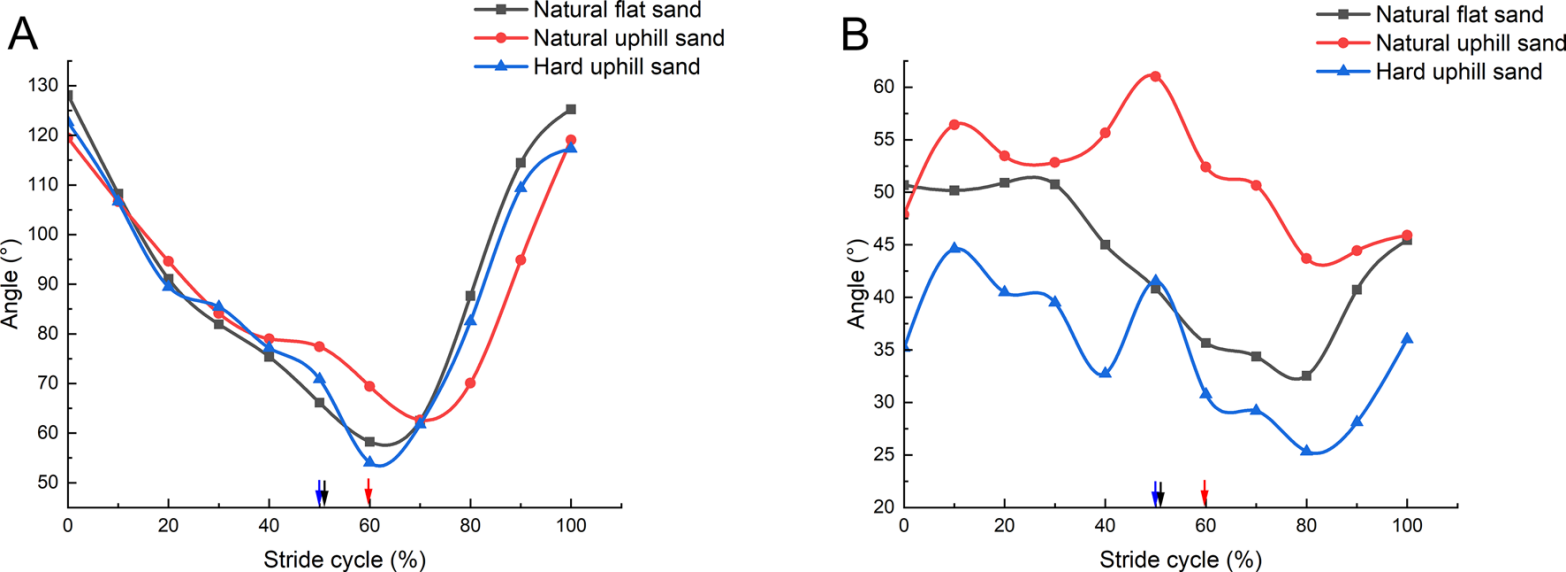
Effect of different sand grounds on continuous joint angles. (**A**) Knee variation pattern effected by locomotion on natural flat sand ground, natural uphill sand ground, and hard uphill sand ground; (**B**) Hip variation pattern effected by locomotion on natural flat sand ground, natural uphill sand ground, and hard uphill sand ground. (24 stride cycles were analyzed for each condition. Arrows denote the transition from the touch-down to the swing phase.)

Figure 8A illustrates that the knee angle of the mallard decreases progressively during the stance and early swing phases, reaching a minimum during the swing phase, and then sharply increases, returning to its initial angle upon re-touchdown. The pattern of knee angle variation is consistent across different sand grounds. Notably, on natural uphill sand ground, the curve showing continuous changes in knee angle exhibits a rightward shift, and the range of knee flexion angle changes is reduced. Figure 8B reveals distinct trends in the continuous variation of hip angle between mallard locomotion on uphill and flat sand grounds. On uphill sand ground, the hip angle initially increases, then decreases during the stance phase, and rapidly ascends to its maximum value at lift-off. During the swing phase, the hip angle gradually declines, then increases, returning to its initial value at the next touch-down. Conversely, on natural flat sand ground, the hip angle remains steady early in the stance phase, decreases progressively during the middle stage, and hits a minimum in the swing phase before returning to its original value in preparation for the next touch-down. The hip angle variation curve on natural uphill sand ground is situated above that on hard uphill and natural flat sand grounds, indicating a broader range of hip angle variation on the natural uphill sand ground.

In summary, when moving on natural sand ground, the TMTPJ, ITJ, and knee of the mallard exhibit a rightward shift in their locomotion patterns. This shift indicates that a decrease in the hardness of the sand ground and an increase in slope cause a greater lag in adjusting the TMTPJ, ITJ, and knee. When moving on uphill sand ground, the mallard experiences two abrupt increases in hip angle during the stance phase, which quickly revert to the initial value after peaking. This pattern is not observed on flat sand ground, suggesting specific adaptations in the hip joint during the stance phase on uphill sand ground. As Table 3 indicates, a reduction in sand ground hardness and an increase in slope lead to decreased flexion angles of the TMTPJ, knee, and hip, accompanied by an increased flexion angle of the ITJ.

**Table 3:** Values and range of changes of hindlimb joint angles at different times under different sand ground.

Sand ground		Natural flat sand		Natural uphill sand		Hard uphill sand	
Period		Stance	Swing	Stance	Swing	Stance	Swing
TMTPJ angles (°)	Initial	164.2	158.9	165..5	163.8	157.4	161.8
	Mid-term	130.6	139.0	132.3	134.7	128.2	130.0
	Range	132.0–164.2	135.5–166.0	134–165.5	134.8–164.7	130.9–157.4	133.8–155.2
ITJ angles (°)	Initial	141.5	148.0	124.4	138.8	145.6	158.9
	Mid-term	144.3	82.3	138.4	77.2	149.2	80.5
	Range	138.3–149	84.6–151.1	123.9–156.3	80.9–130.5	141.7–158.1	84.8–146.8
Knee angles (°)	Initial	128.1	67.8	119.5	69.9	122.7	68.5
	Mid-term	85.5	73.8	83.0	69.3	87.0	69.9
	Range	66.1–128.1	58.2–125.2	69.4–119.5	62.7–119.1	70.9–112.7	54.1–117.3
Hip angles (°)	Initial	55.2	46.9	50.5	55.1	46.9	43.2
	Mid-term	45.4	35.9	54.0	44.5	43.3	29.6
	Range	40.8–50.9	32.5–45.4	47.9–61.0	43.7–50.7	32.7–44.6	25.3–36

The joint angle value at the lift-off is the initial value during the swing phase.

## Discussion

### Analysis of the effect of hindlimb locomotion on mallards

Mallards demonstrate distinct adjustments in hindlimb joint angles during the stance phase, contingent on the hardness and slope of the sand ground they are moving on. Analysis reveals that mallards on natural and hard sand ground exhibit increased duty factors correlating with reduced ground hardness and escalated slope (Table 1). When the ground hardness decreases and the slope increases, mallards adjust by reducing their stride length and prolonging their stance phase duration. This decrease in stride length significantly diminishes the mallards' speed of locomotion. Our findings are consistent those of previous studies^1^.

As the slope remains constant, a decrease in the hardness of the sand ground leads to increased flexion of the TMTPJ and ITJ at touch-down, with the ITJ showing increased flexion at mid-stance and lift-off. The knee experiences minimal effect, while the hip shows less flexion during mid-swing. Delayed adjustments in the TMTPJ, ITJ, and knee lead to an elevated duty factor and extended stance phase duration.

Conversely, when ground hardness is consistent, an increase in slope heightens the flexion of the ITJ at touch-down, with no significant effects on the TMTPJ, knee, or hip at any phase. At mid-swing, all joint angles exhibit no notable differences between uphill and flat sand grounds, suggesting that an 8°–10° slope does not affect the clearance space of the mallard's hindlimb. Delayed adjustments in the TMTPJ, ITJ, and knee joints result in an increased duty factor and prolonged stance phase duration. Continuous joint angle analysis of the hip reveals that, during moving on uphill grounds, the hip joint briefly extends at stance phase, a pattern absent on flat sand ground. This finding indicates a greater involvement of the hip in adjusting the hindlimb posture when mallards move on uphill sand grounds.

### Analysis of the strategies and functions of mallard hindlimb locomotion

Mallards adeptly adjust to varied sand grounds through coordinated locomotion of their hindlimbs, where each joint assumes distinct postures for optimal adaptation. The unique functionality of each joint plays a crucial role in achieving harmony in hindlimb locomotion.

The TMTPJ initially flexes at the onset of stance, facilitating sequential contact of the toes with the ground. As the stance phase progresses, the TMTPJ gradually extends; this extension helps to keep the toes in contact with the ground as the body passes over the hindlimbs. During the swing phase, the TMTPJ flexes to ensure adequate ground clearance and then swiftly extends, readying the foot for the subsequent stance. An increased extension angle of the TMTPJ at the initial stance phase pre-empts the landing of the foot, thereby extending the stance phase duration. Furthermore, mallards adjust the TMTPJ flexion angle to modify the landing angle, catering to variations in sand ground particle size and thickness^27^.

The ITJ maintains a relatively stable angle during the early phase of ground stance, providing necessary support for the hindlimb. Its extension in the latter part of the stance phase propels the hindlimb off the ground, mirroring the ITJ angle variation pattern observed in magpies^8^. The ITJ rapidly decreases in the early swing phase, facilitating swift ground clearance for hindlimb locomotion. Rapid extension in the later swing phase aligns the toes for stance during forward extension of the hindlimb, effecting stride length at stance. Additionally, when animals run at higher speeds, the oscillations between potential and kinetic energy are thought of in terms of being in phase^17,27,32,33^. Hence, a shorter stride length may be advantageous in uphill locomotion or on softer ground, minimizing changes in the body's center of mass and enhancing locomotion smoothness.

The knee extension is crucial for propelling the mallard's hindlimb forward, significantly affecting stride length. However, this experiment did not observe any notable difference in the knee's role across various sand grounds. The mallard's relatively high locomotion speed in natural environments may explain the lack of a notable difference in the knee's role across various sand grounds, indicating that the knee's capacity to regulate stride length may have reached its maximum potential. Therefore, the mallard primarily manages stride length adjustments through ITJ flexion, enabling adaptation to diverse sand grounds.

The hip joint, being the largest and most stable in the body, supports the mallard's weight with the hindlimbs. A relatively stable hip with rapid extension has been seen elsewhere, such as in guineafowl^11^, in which it maintains a consistently flexed position throughout the stride cycle, exhibiting minimal variation. Notably, during the stance phase on uphill ground, the hip experiences brief extensions, a pattern somewhat akin to the hip locomotion observed in ostriches and *Dipsosaurus dorsalis*. In ostriches, following toe touchdown, the hip briefly extends and then flexes, extending rapidly towards the end of the stance phase, potentially aiding in energy storage and release^16^. Similarly, *Dipsosaurus dorsalis* increases pelvic rotation during uphill locomotion for enhanced propulsion^25^. Thus, we surmise that the hip's extension during the stance phase significantly contributes to the mallard's locomotion efficiency.

The TMTPJ of the mallard hindlimb is crucial in controlling contact angles and adjusting the timing of the stance phase on natural sand grounds. Meanwhile, the ITJ and knee joints play vital roles in increasing stride length, and the hip joint distinctively aids in propulsion. Through the synergistic coordination of these four joints, mallards achieve seamless mobility on both soft and inclined grounds.

## Conclusion

In this study, comparative experiments were conducted on mallards navigating three different natural sand grounds: natural flat, natural uphill, and hard uphill sand ground. The results demonstrate a clear adaptive response of mallards to variations in slope and ground hardness, characterized by a reduction in stride length and an increase in the duty factor. Instantaneous joint angle analysis revealed that an increase in ground slope resulted in decreased flexion of the ITJ at touch-down. Conversely, a decrease in ground hardness leads to increased flexion of the ITJ and decreased flexion of the TMTPJ at touch-down. Continuous joint angle analyses indicated that both a decrease in ground hardness and an increase in slope caused a more pronounced delay in adjusting the TMTPJ, ITJ, and knee throughout the entire stride cycle of the mallards. Notably, a brief extension of the hip joint during the stance phase was observed to significantly enhance propulsion when moving on uphill sand ground. The study found that the primary effect of different sand ground conditions on mallard locomotion was reflected in the variation of flexion and extension degrees in the hindlimb joints. In response to softer ground and slopes, mallards increased the flexion angle of the ITJ to shorten stride length. Simultaneously, they reduced the flexion of the TMTPJ and slowed joint adjustments, thereby extending the duration of stance. These modifications enabled mallards to maintain locomotion stability within each stride cycle through shorter stride length and extended stance time. This study explores the effect of different slopes and hardness ground on the hindlimb locomotion of mallards. It underscores the significant challenges encountered by the hindlimb of mallards on diverse natural grounds and delineates the proactive adaptive strategies they employ.

### Supplementary Information

Supplementary Information 1.

Supplementary Information 2.

## Data Availability

All data generated or analysed during this study are included in Supplementary Information files.
