# Supplementary material for: Mallard hindlimbs locomotion system respond to changes in sandy ground hardness and slope
Source: Sci Rep. 2024 Jul 5;14:15536. doi: 10.1038/s41598-024-66181-z (PMC11226611; doi:10.1038/s41598-024-66181-z)

**Ethic Note**

The experiments in this study comply with the current laws of China. This study did not involve any animal capture or sampling of blood or tissue. During the experiment, only allow the mallard to actively participate in the walking process and if any mallard exhibits abnormal locomotion, the experiment will be immediately halted. Custom-designed duck cages were used to house the mallards, ensuring their natural and healthy living conditions by providing them with adequate water and nutrition. After the experiment was completed, mallards returned to the farm healthy and safe. The Animal Protection and Use Committee of Jilin University, China, approved the living and experimental conditions of the samples (reference No. SY202206100).


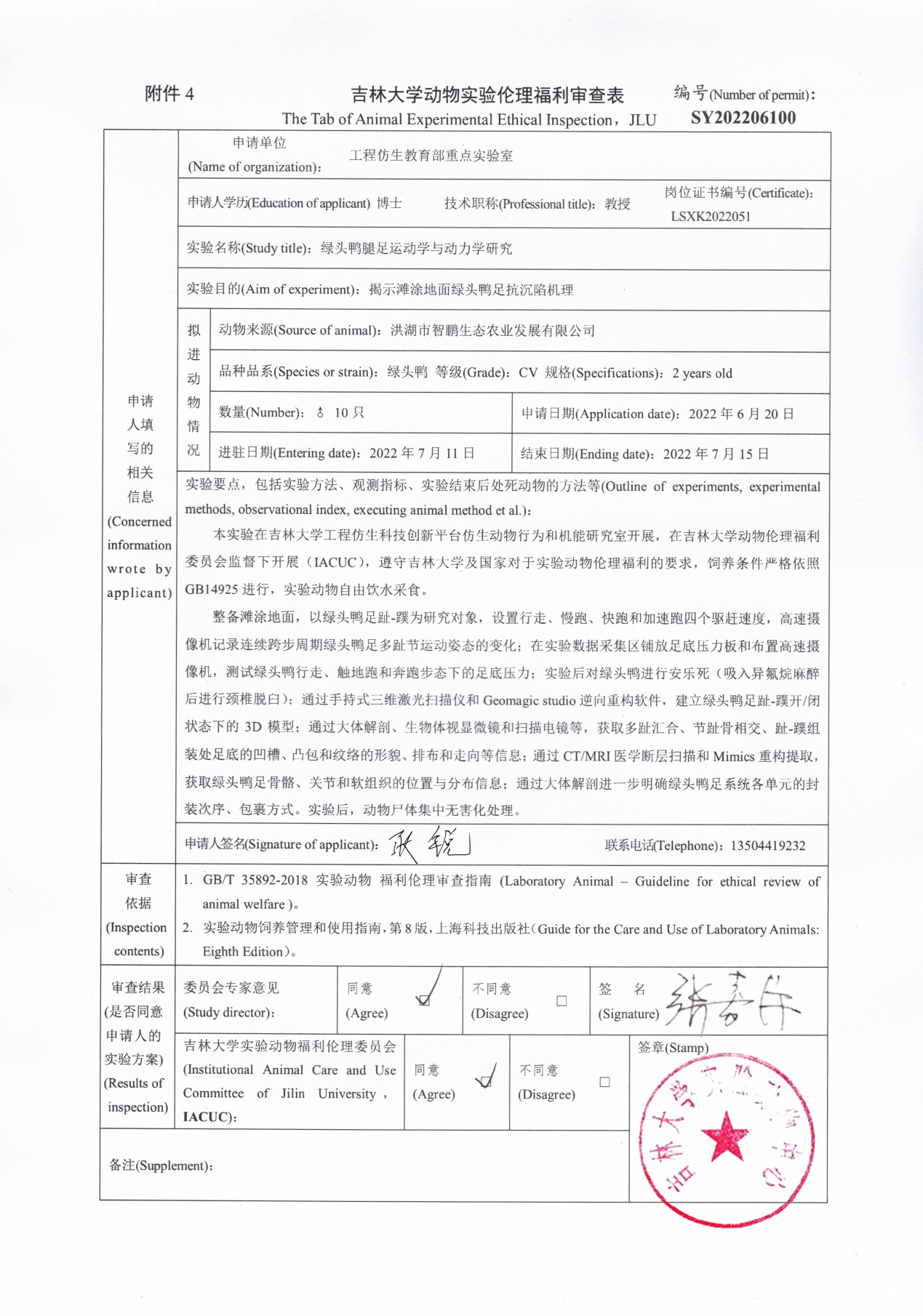

Supplement: Supplementary file 1 — Supplementary Information 1. [file 41598_2024_66181_MOESM1_ESM.docx]
